# Modified aging of elite athletes revealed by analysis of epigenetic age markers

**DOI:** 10.18632/aging.101385

**Published:** 2018-02-15

**Authors:** Magdalena Spólnicka, Ewelina Pośpiech, Jakub Grzegorz Adamczyk, Ana Freire-Aradas, Beata Pepłońska, Renata Zbieć-Piekarska, Żanetta Makowska, Anna Pięta, Maria Victoria Lareu, Christopher Phillips, Rafał Płoski, Cezary Żekanowski, Wojciech Branicki

**Affiliations:** 1 Central Forensic Laboratory of the Police, Warsaw, Poland; 2 Malopolska Centre of Biotechnology of the Jagiellonian University, Krakow, Poland; 3 Department of Theory of Sport, Józef Pilsudski University of Physical Education in Warsaw, Warsaw, Poland; 4 Forensic Genetics Unit, Institute of Forensic Sciences, University of Santiago de Compostela, Santiago de Compostela, Spain; 5 Laboratory of Neurogenetics, Department of Neurodegenerative Disorders, Mossakowski Medical Research Centre, Polish Academy of Sciences, Warsaw, Poland; 6 Department of Medical Genetics, Centre for Biostructure, Medical University of Warsaw, Warsaw, Poland; 7 Department of Rehabilitation, Physiotherapy Division, Medical University of Warsaw, Warsaw, Poland

**Keywords:** elite athletes, intense physical exercise, aging, epigenetic age, DNA methylation, *TRIM59*, *KLF14*

## Abstract

Recent progress in epigenomics has led to the development of prediction systems that enable accurate age estimation from DNA methylation data. Our objective was to track responses to intense physical exercise of individual age-correlated DNA methylation markers and to infer their potential impact on the aging processes. The study showed accelerated DNA hypermethylation for two CpG sites in *TRIM59* and *KLF14*. Both markers predicted the investigated elite athletes to be several years older than controls and this effect was more substantial in subjects involved in power sports. Accordingly, the complete 5-CpG model revealed age acceleration of elite athletes (*P*=1.503x10^-7^) and the result was more significant amongst power athletes (P=1.051x10^-9^). The modified methylation of *TRIM59* and *KLF14* in top athletes may be accounted for by the biological roles played by these genes. Their known anti-tumour and anti-inflammatory activities suggests that intense physical training has a complex influence on aging and potentially launches signalling networks that contribute to the observed lower risk of elite athletes to develop cardiovascular disease and cancer.

## Introduction

Aging is a complex process associated with various molecular modifications in cells including epigenetic transitions that create changes in gene expression. Many studies have indicated DNA methylation (DNAmet) is an important component of aging. In addition to inherited, genetically driven DNAmet changes, a broad spectrum of environmental factors can influence the methylation status of many sites making the human DNA methylome a sensitive marker set of epigenetic drift rather than a stable imprint [1–3]. Therefore, age predicted with epigenetic markers better reflects the biological age than the chronological age of an individual and such differences in aging rates may potentially be linked to various clinical and environmental factors.

With the advent of high-throughput sequencing technologies epigenome-wide association studies became available and led to successful identification of age-correlated DNAmet changes [1,2,4–8]. These discoveries were associated with development of age prediction models that have allowed unprecedented accuracy for age estimation regimes. Epigenetic age prediction methods have been mostly developed based on DNA extracted from blood but other DNA sources have also been investigated [9]. The studies of age-related DNAmet markers have also revealed that age acceleration defined as the discrepancy between methylation age and chronological age, may be a reliable indicator of increased disease and mortality risk [10–13]. Since age acceleration can be a biologically meaningful biomarker, knowledge of its progression may find practical application in many disciplines including sports medicine and forensics [14,15].

It is widely accepted that people who engage in moderate physical activity will benefit from better mood and cognition, lower levels of cardiovascular disease and a slower rate of aging [16,17]. Indeed, the bulk of research has reported longer life expectancy and lower mortality risk for elite athletes compared to the general population and such results are particularly consistent for groups of endurance athletes. Results from several large meta-analyses are the most informative [18–20]. Investigating the epigenome of elite athletes provides a potentially informative way to study how life-style can affect the functioning of the human genome. Moreover, it has been shown that DNAmet in various CpG sites may undergo diverse regulation [21]. Thus, the tracking of individual epigenetic markers may provide better insight into the molecular processes that underlie aging by disclosing the cellular activity and potential interactions the key genes are involved in. The possibility to examine more closely the molecular processes associated with aging from the advent of epigenomic analysis and epigenetic age prediction, permits more objective exploration of the role of physical activity in a healthy lifestyle.

Our study aimed to examine whether intense physical exercise can alter DNAmet profiles and lead to changes in the rate of aging. To do this we studied the DNAmet profile of Polish professional athletes at 5 CpG sites in five different genes that we had previously selected and developed into a DNAm-based age prediction calculator [22]. The study intended to distinguish markers following a regular clock-like pattern of methylation change from those sites more prone to environmental factors associated with the life of top-level athletes.

## RESULTS

DNA methylation status at the 5 CpG sites in five age related genes was analyzed in 176 athletes, in comparison to 128 healthy control individuals from the general population matched by age (*P*=0.760) and gender (*P*=0.949). Elite athletes comprised young individuals with a mean age of 24.1 and was 112 males (63.6%) and 64 females (36.4%); as outlined in Table 1. The group of power athletes consisted of 57 males (60.6%) and 37 females (39.4%) and had a mean age of 23 years. The group of endurance athletes comprised 55 males (67.1%) and 27 females (32.9%) with a mean age of 25.2 years. The control group had a mean age of 24.2 and was 81 males (63.3%) and 47 females (36.7%). Age distribution graphs for athletes and controls by gender are provided in Supplementary Figure 1. The mean age of males in the group of athletes was higher (24.9) compared to females (22.7); and similarly in the control group with a mean age of 25.4 for males and 22.2 for females.

**Table 1:** DNA methylation status of single age-related CpG sites measured in Polish professional athletes compared to age- and gender-matched controls from the general population

Locus CpG site	Mean % of DNAmet		*P* value	Mean % of DNAmet		*P* value	Mean % of DNAmet		*P* value
	All athletes	Controls		Endurance	Controls		Power	Controls	
*ELOVL2* C7	60.89	61.09	0.791	61.94	61.09	0.342	59.98	61.09	0.196
*MIR29B2C* C1	79.43	80.54	0.113	80.04	80.54	0.542	78.89	80.54	0.058
*FHL2* C2	36.37	35.87	0.375	36.41	35.87	0.453	36.33	35.87	0.465
***TRIM59* C7**	**31.43**	**28.19**	**1.894x10^-7^**	**30.56**	**28.19**	**0.004**	**32.18**	**28.19**	**2.946x10^-7^**
***KLF14* C1**	**5.16**	**4.12**	**8.974x10^-10^**	**5.23**	**4.12**	**1.866x10^-7^**	**5.10**	**4.12**	**4.987x10^-7^**

. Significant values are marked in bold.

### DNA methylation status

The study revealed significantly altered DNA methylation patterns between the whole group of athletes and controls in *TRIM59* (*P*=1.894x10^-7^) and *KLF14* (*P*=8.974x10^-10^), as well as separately for endurance and power athletes (Table 1). Although the results were significant for both endurance and power athletes, a more marked difference was noted in *TRIM59* C7 for the power athletes (P=2.946x10^-7^). In *KLF14* C1, hypermethylation was observed for power and endurance athletes with a similar level of significance of *P*=4.987x10^-7^ and *P*=1.866x10^-7^, respectively (Table 1). Analyses performed separately for females and males confirmed *TRIM59* and *KLF14* markers to be significantly hypermethylated in athletes of both genders. (Supplementary Table 1).

### Age prediction and age acceleration

Table 2 shows that our established multivariate 5-CpG ANN model predicted the whole group of elite athletes with a lower accuracy of MAE = 3.3, compared to healthy controls of similar mean age and age distribution that had a MAE = 2.4, *P* = 3.815x10^-4^. When the two athlete groups were analysed separately, a significant decrease of age prediction accuracy from MAE=2.4 to MAE = 3.7 was only found for power athletes (*P*=5.727x10^-5^). Analyses performed separately for females and males confirmed reduced accuracy of age prediction in the group of power athletes vs. controls in both genders (*P*=0.013 and *P*=0.002, for females and males respectively); outlined in Supplementary Table 2.

**Table 2:** Age prediction accuracy measured by MAE using the 5-CpG sites model published in [22], modified by using ANN statistical analysis.

Model	Compared groups	N	MAE	Std. Deviation	*P* value
5 CpG model [22]	Athletes	175	3.273	2.620	**3.815x10^-4^**
	Controls	128	2.361	1.797	
	Endurance	82	2.776	2.391	0.180
	Controls	128	2.361	1.797	
	Power	93	3.712	2.745	**5.727x10^-5^**
	Controls	128	2.361	1.797	

One sample was removed from prediction analysis because of missing data in *MIR29B2C* C1.

Age acceleration from chronological and methylation age divergence (determined using the 5-CpG model) was further calculated and tested for its association with intense physical exercise. This analysis showed the status of elite athletes is significantly associated with age acceleration (*P*=1.503x10^-7^) and was significant for both groups (1.051x10^-9^ and 0.004 for power and endurance athletes, respectively; Table 3). Analysis performed at the level of single CpG predictors showed that significant association of the status of elite athletes with age acceleration arose from increased DNA methylation of the CpGs at *TRIM59* and *KLF14*. The level of significance of association between the status of power athletes and age acceleration was highest when just *TRIM59* was used as a predictor of age (*P*=1.239x10^-10^ comparing to *P*=0.003 obtained for endurance athletes), but at a comparable level in *KLF14* in both groups (*P*=3.345x10^-7^ and *P*=2.116x10^-8^, for endurance and power groups, respectively). The type of sport competition performed was also found to be significantly associated with age acceleration (*P*=0.003) indicating the increased aging of athletes engaged in power sports compared to endurance sports (Table 3).

**Table 3:** Association between extensive physical exercise and age acceleration in Polish athletes.

Models	Age acceleration (5 CpGs model)			Age acceleration (*KLF14* C1)			Age acceleration (*TRIM59* C7)		
	Effect size*	SE	*P* value	Effect size*	SE	*P* value	Effect size*	SE	*P* value
Athletes vs. Controls	2.156	0.401	**1.503x10^-7^**	5.559	0.845	**2.111x10^-10^**	4.629	0.834	**6.270x10^-8^**
Endurance vs. Controls	1.329	0.452	**0.004**	5.478	1.039	**3.345x10^-7^**	2.647	0.867	**0.003**
Power vs. Controls	2.885	0.452	**1.051x10^-9^**	5.630	0.968	**2.116x10^-8^**	6.194	0.917	**1.239x10^-10^**
Power vs. Endurance	**1.665**	**0.558**	**0.003**	-0.052	1.169	0.965	**3.455**	**1.272**	**0.007**

*Effect sizes are the beta coefficients from linear regression models adjusted for age (years) and gender and additionally for training experience (years) when the type of the competition (power vs. endurance) was analysed.

**Figure 1:**
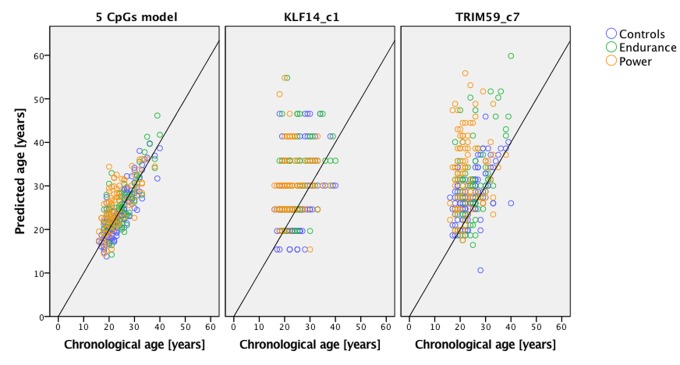
**Predicted age vs. chronological age of power and endurance athletes compared to age- and gender-matched controls.** Predictions based on the 5 CpGs model and separately *KLF14* c1 and *TRIM59* c7.

**Figure 2:**
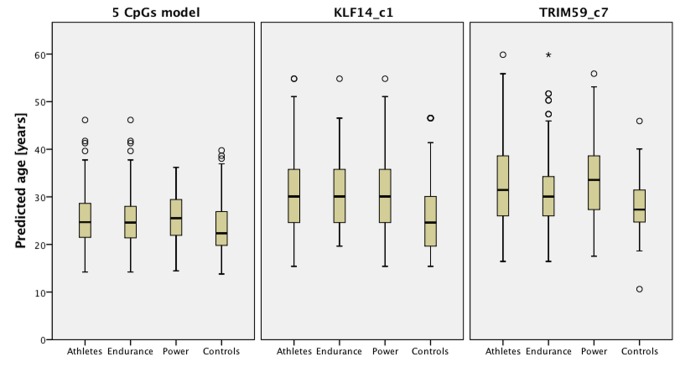
**Predicted age of athletes comparing with age- and gender- matched controls based on predictors from 5 CpGs model, *KLF14* and *TRIM59***.

Predicted age versus chronological age in endurance and power athletes plus the group of controls is presented on Figure 1, which shows predictions based on the 5-CpGs model and *TRIM59* and *KLF14* separately. Figure 2 shows that age prediction based on CpG site C1 in *KLF14* indicated athletes to be an average 5.5 years older than healthy controls (*P*=1.766x10^-9^), endurance athletes to be 5.9 years older than controls from the general population (*P*=2.511x10^-7^) and power athletes to be 5.1 years older than healthy controls (*P*=8.443x10^-7^). Age prediction performed with DNA methylation values from CpG site C7 in *TRIM59* showed athletes to be an average 4.5 years older than healthy controls (*P*=9.405x10^-8^), power athletes to be 5.6 years older than healthy controls (*P*=2.105x10^-7^) while endurance athletes were predicted to be 3.3 years older than healthy controls (*P*=0.003).

## DISCUSSION

### The impact of physical exercises on epigenetic age

Very intensive physical exercise as a routine part of elite athlete training has been shown to have favorable effects on lifespan longevity and is associated with a lowered risk of cardiovascular diseases and cancer in later life [19,20,24]. Importantly, individuals with active lifestyles but free from the stress of extreme exercise regimes benefit from regular physical activity by reducing their risk of all forms of mortality, and in proportion to the intensity of the training [25]. Better insight into the molecular processes behind the aging of elite athletes is now possible thanks to recent advances in epigenetics and the efficiency of DNAmet age prediction systems in providing reliable indicators of the aging rate. Our study indicates accelerated aging of the whole group of elite athletes, with power athletes shown to have higher levels of age acceleration compared to endurance athletes. The observed acceleration of aging may potentially have significant implications, as it has been shown that a difference between the chronological age of an individual and the age measured by DNA methylation is an indicator of a person’s overall condition and is predictive of mortality in later life.

### The meaning of epigenetic age

The epigenetic clock CpGs defined as correlated with age consistently between individuals and included in various age prediction models, reveal some degree of inter-individual variability [26]. There is an on-going debate about the real meaning of age measured by DNAmet status. The picture arising from recent research indicates that various markers included in age estimation models may be regulated in a different way. Particular age-correlated CpGs could be liable to moderation by inherited factors and the environment [2]. It has been shown that the epigenetic clock developed by Horvath can also measure age in non-proliferative tissues but at the same time there is evidence that hypermethylation of one of the most informative age predictors – *ELOVL2*, is associated with cell replications and thus indicates a mitotic age [21]. Therefore, it is conceivable that some DNAmet markers may be more sensitive to the environment than others and consequently they can provide a better measure of epigenetic drift. Consequently, it is unsurprising that not all age predictors are informative in terms of increased mortality risk. The study of Lin et al selected 37 CpGs indicative of life expectancy from their full set of 99 markers. Additionally, they tested alternative age prediction models and found that the marker sets proposed by Horvath and Hannum contained mortality sensitive CpG sites [12,1,2]. Two out of five markers investigated here, namely *TRIM59* and *KLF14*, are among the suggested life expectancy-sensitive markers from the Hannum model. *TRIM59* belongs to the ten most informative markers from the age predictors included in that age prediction model [12]. Notably, an independent study has demonstrated that 5-year higher DNA methylation age predicted by the Hannum model was associated with a 21% increased mortality risk [11]. Overall, multiple studies have reported an 11% to 35% increase in mortality risk for individuals with a 5-year higher DNA methylation age [11,12,27]. Recently, Zhang et al presented a list of life expectancy DNA methylation markers which are independent from the epigenetic clock, providing further evidence that aging related markers can be different from markers predicting chronological age [13].

### The molecular role of individual age-correlated DNA methylation markers

More detailed analysis of the accelerated aging of elite athletes indicated by our model revealed that accelerated hypermethylation of *TRIM59* and *KLF14* were the principal cause of the changes. Both, are widely replicated age predictors used in various age estimation models based on DNA methylation analysis [2,22,28,29]. Most studies have indicated longer life expectancy in top athletes compared to the general population and our finding of an accelerated epigenetic age of elite athletes seems to contradict these results. However, careful inspection of the molecular nature of *TRIM59* and *KLF14* might reveal their specific impact on aging processes in these athletes. *TRIM59* has recently emerged as a powerful oncogene involved in induction of cellular senescence. Valiyeva and colleagues have shown that *TRIM59* can affect Ras and RB signal pathways and its knockdown leads to S-phase cell cycle arrest and cell growth retardation [30]. Importantly, it has been shown that interaction between Ras and pRb may launch cellular processes leading to cellular senescence and tumor suppression [31]. There is growing evidence that *TRIM59* is a promoter of cell proliferation and migration. Increased expression of this gene has been reported in various tumors [32–35]. In contrast to the initial perception that cellular senescence is harmful, it has been proposed to have beneficial biological implications. Cellular senescence acts as a tumour-suppressive mechanism that irreversibly blocks cellular proliferation in response to stress and through this process, creates a barrier to the development and progression of cancer. Moreover, this apparent cancer moderation is accompanied by the acquisition of a senescence-associated secretory phenotype that induces cell plasticity which is important in tissue regeneration or wound healing [36]. Thus, hypermethylation of *TRIM59* indicates accelerated aging of elite athletes, especially involved in power sports but at the same time it may have an anticancer effect. Interestingly, a study of mutants in the p53 tumor suppressor gene has shown that increased tumor protection occurs from modified p53 protein, while at the same time these induce early onset aging in mice; linking senescence, aging and cancer [37]. Our previous study revealed aberrant hypermethylation of *TRIM59* in early-onset Alzheimer’s disease patients and in Graves’ disease patients [23]. This seems to further support the hypothesis of the involvement of *TRIM59* in cellular senescence since chronic neuro-inflammation has been shown to be important in aging and neuro-degeneration, while autoimmunity is known to induce inflammation, tissue damage and remodelling [38,39].

The observed hypermethylation of *KLF14* also indicates accelerated aging of elite athletes. Recent findings showing the role of *KLF14* in chronic inflammatory responses and the pathogenesis of atherosclerosis may be particularly relevant to this finding [40,41]. Wei et al showed that *KLF14* may be involved in induction of inflammation and for this reason knockdown of this gene reduces pro-inflammatory cytokines and the formation of atherosclerotic lesions. This anti-inflammatory effect was suggested to prevent atherosclerosis: a known chronic inflammatory disease [40]. It is interesting to speculate whether DNA methylation of *TRIM59* is involved in the process of cellular senescence and the associated beneficial biological effects. Similarly, the hypermethylation of *KLF14* could have an anti-inflammatory role preventing cardiovascular disease. These hypotheses deserve further investigation, but they may already explain the increased life expectancy of elite athletes and in particular, the lower risk they have to develop cancer and cardiovascular diseases [19].

## CONCLUSIONS

Our study findings indicate various age predictors can show detectable differences in sensitivity to intense physical activity. The study revealed accelerated hypermethylation of *TRIM59* and *KLF14* in elite athletes suggesting their higher epigenetic age, which is contrary to epidemiological data showing longer life duration of athletes. However, the two genes are involved in anti-tumor and anti-inflammatory activities, respectively, which in turn could affect better life-expectancy. The results obtained may reveal a complex influence of intense physical training on the aging process. We speculate that the resulting diminished expression of these genes may potentially launch signaling networks that contribute to an observed lower risk for elite athletes to develop cardiovascular disease and cancer.

Investigation of an extended list of age-related markers and detailed analyses of the relevance of individual CpGs for biological aging will aid in the understanding of the role of DNA methylation in human aging and an assessment of the relevance of DNA methylation as an indicator of the condition of an individual.

## MATERIALS AND METHODS

### Samples and analysis of DNA methylation

The study was approved by the Ethics Committee of the Józef Piłsudski University of Physical Education in Warsaw (SKE 01-50/2012) and the institutional ethical review board of the Ethics Committee of Investigation in Galicia, Spain (CAEI: 2013/543). Written informed consent was obtained from the elite athletes and counterpart controls from the general population forming the study subjects. Peripheral blood was collected in EDTA tubes from 176 elite athletes in the years 2010-2014. Chronological age at the time of blood collection was used for all the calculations in this study. Top-level professionals (including medallists of Olympic Games and World championships) in power and endurance sports, 100 athletics, 21 speed skaters, 28 swimmers and 27 rowers were enrolled in the study. 128 blood samples from the general population matched by age and gender, were recruited for the control group (Table 4). DNA was extracted using standard methods and quantified using Qubit dsDNA High Sensitivity Assay Kit (Thermo Fisher). From 500 ng to 2 μg of DNA was treated with sodium bisulfite using the EpiTect 96 Bisulfite Kit (Qiagen, Hilden, Germany), following manufacturer’s guidelines. Specific CpG sites were analysed using previously described primers and methods [22]. Details of the analysed sites are presented in Table 5.

**Table 4:** Characteristics of the Polish elite athletes and controls used in the study.

Sport discipline	N [%]	The specifics of competition		Male N [%]			Mean Age ± SD		
		Power N [%]	Endurance N [%]	All	Power N [%]	Endurance N [%]	All	Power N [%]	Endurance N [%]
Athletics	100 [56.8]	70 [70.0]	30 [30.0]	61 [61.0]	42 [60.0]	19 [63.3]	24.8 ±5.3	23.8±4.9	27.2±5.3
Speed skating	21 [11.9]	7 [33.3]	14 [66.7]	17 [81.0]	6 [85.7]	11 [78.6]	21.5 ± 4.1	19.3±1.4	22.6±4.6
Swimming	28 [15.9]	17 [60.7]	11 [39.3]	16 [57.1]	9 [52.9]	7 [63.6]	22.0 ± 5.0	21.4±3.8	23±6.5
Rowing	27 [15.4]	0 [0.0]	27 [100.0]	18 [66.7]	-	18 [66.7]	25.4 ± 4.2	-	25.4±4.2
Total athletes	176 [100]	94 [53.4]	82 [46.6]	112 [63.6]	57 [60.6]	55 [67.1]	24.1 ± 5.1	23.0±4.8	25.2±5.3
Controls	128 [100]	-	-	81 [63.3]	-	-	24.2 ± 5.0	-	-

**Table 5:** The studied markers and CpG sites.

Gene locus	CpG site	Chromosome	Chromosome location (GRCH38)
*ELOVL2*	C7	Chr6	11044634
*MIR29B2C*	C1	Chr1	207823681
*TRIM59*	C7	Chr3	160450199
*KLF14*	C1	Chr7	130734355
*FHL2*	C2	Chr2	105399288

### Statistical analyses and prediction of DNA methylation age

Analyses were performed using PS IMAGO 4 (IBM SPSS Statistics 24). DNA methylation was measured in the two groups of 176 elite athletes plus 128 healthy counterparts of the age/gender matched control group at 5 CpGs included in the age prediction model (Table 5). Methylation data was analysed using independent sample Student’s *t* test and the balanced distribution of age in the athlete and control groups confirmed with nonparametric Kolmogorov-Smirnov test. Age predictions were made with the method developed by Zbieć-Piekarska et al. [22] after remodelling with the artificial neural networks (ANN) approach applied in the study of Spólnicka et al. [23]. The training set of 305 individuals from the above previous studies was used for the age predictions of the current study. The predicted age of athletes was compared with their true chronological age to calculate mean absolute error (MAE). Independent sample Student’s *t* test was used to compare mean predicted age and MAE. Age acceleration (AA) - a measure of the discrepancy between methylation age and chronological age - was designated in the form of residuals calculated from linear regression analysis; where predicted age was treated as the dependent variable and chronological age as the independent variable as described in [14]. Association analysis of individual levels of intense training with age acceleration was tested using linear regression, controlling for age and gender and in some analyses, for the number of years spent in training.

## Supplementary Material

Supplementary File
