# Supplementary material for: Modified aging of elite athletes revealed by analysis of epigenetic age markers
Source: Aging (Albany NY). 2018 Feb 15;10(2):241–52. doi: 10.18632/aging.101385 (PMC5842850; doi:10.18632/aging.101385)
Supplement: Supplementary File [file aging-10-101385-s001.pdf]

## SUPPLEMENTARY MATERIAL

A) Age distribution in the studied group of all athletes

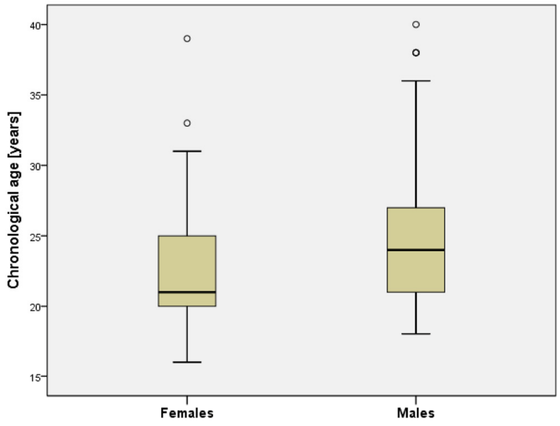


B) Age distribution in the studied group of endurance athletes

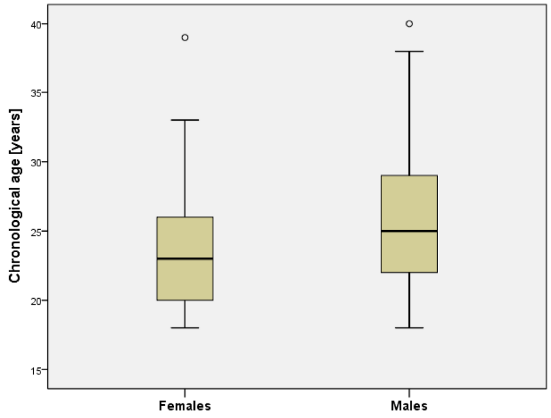


C) Age distribution in the studied group of power athlet

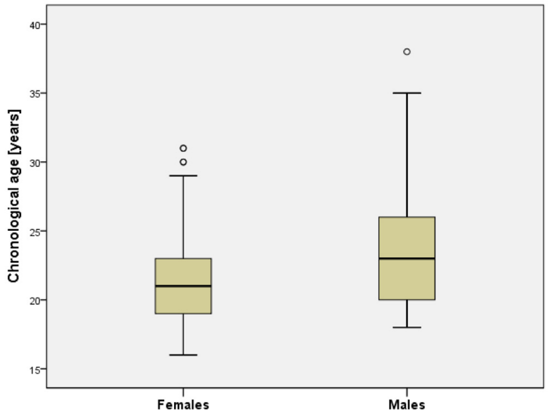


D) Age distribution in the control group

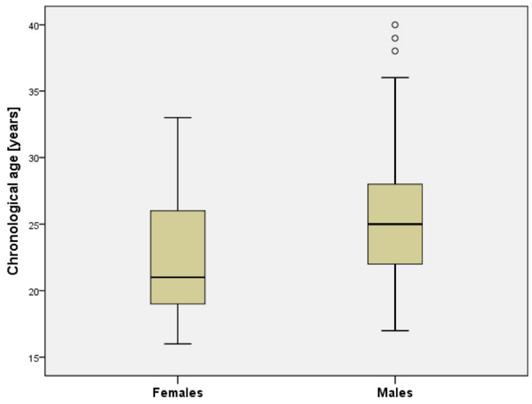


**Supplementary Figure 1.** Distribution of age in the studied group of athletes divided by gender of the individuals.

**Supplementary Table 1. DNA methylation status of single age-related CpG sites measured in Polish athletes divided by gender and compared to age-matched controls from the general population.**

| Locus CpG site | Mean % of DNAmet | | *P* value | Mean % of DNAmet | | *P* value |
|---|---|---|---|---|---|---|
| | Females | | | Males | | |
| | Athletes (N=64) | Controls (N=47) | | Athletes (N=112) | Controls (N=81) | |
| *ELOVL2* C7 | 59.22 | 58.87 | 0.754 | 61.85 | 62.37 | 0.573 |
| *MIR29B2C* C1 | 79.86 | 81.45 | 0.179 | 79.19 | 80.01 | 0.341 |
| *FHL2* C2 | 36.44 | 36.89 | 0.647 | 36.33 | 35.27 | 0.122 |
| ***TRIM59* C7** | **31.03** | **27.15** | **$1.144x10^{-5}$** | **31.65** | **28.79** | **0.001** |
| ***KLF14* C1** | **4.84** | **3.96** | **0.002** | **5.34** | **4.21** | **$8.379x10^{-8}$** |

Significant values are marked in bold.

**Supplementary Table 2. Age prediction accuracy measured by MAE using the 5-CpG sites model compared between athletes and controls divided by gender.**

| 5 CpG model | | | | | |
|---|---|---|---|---|---|
| Model | Compared groups | N | MAE | Std. Deviation | *P* value |
| Females | Athletes | 63 | 3.684 | 2.906 | **0.010** |
| | Controls | 47 | 2.501 | 1.822 | |
| | Endurance | 27 | 3.233 | 2.472 | 0.149 |
| | Controls | 47 | 2.501 | 1.822 | |
| | Power | 36 | 4.023 | 3.185 | **0.013** |
| | Controls | 47 | 2.501 | 1.822 | |
| Males | Athletes | 112 | 3.042 | 2.428 | **0.013** |
| | Controls | 81 | 2.280 | 1.789 | |
| | Endurance | 55 | 2.551 | 2.341 | 0.445 |
| | Controls | 81 | 2.280 | 1.789 | |
| | Power | 57 | 3.515 | 2.437 | **0.002** |
| | Controls | 81 | 2.280 | 1.789 | |

One sample was removed from prediction analysis because of missing data in MIR29B2C C1.
